# Research progress on the mechanism of angiogenesis in wound repair and regeneration

**DOI:** 10.3389/fphys.2023.1284981

**Published:** 2023-11-27

**Authors:** Zhuojun Shi, Chong Yao, Yujie Shui, Site Li, Hong Yan

**Affiliations:** ^1^ Department of Plastic and Burns Surgery, The Affiliated Hospital of Southwest Medical University, National Key Clinical Construction Specialty, Wound Repair and Regeneration Laboratory, Luzhou, Sichuan, China; ^2^ Laboratory of Plastic Surgery, Department of Plastic Surgery and Reconstruction, Second Hospital of West China, Sichuan University, Chengdu, Sichuan, China

**Keywords:** mechanisms of angiogenesis regulation, wound healing, regeneration, signal conduction, diseases associated with angiogenesis, capillary

## Abstract

Poor wound healing and pathological healing have been pressing issues in recent years, as they impact human quality of life and pose risks of long-term complications. The study of neovascularization has emerged as a prominent research focus to address these problems. During the process of repair and regeneration, the establishment of a new vascular system is an indispensable stage for complete healing. It provides favorable conditions for nutrient delivery, oxygen supply, and creates an inflammatory environment. Moreover, it is a key manifestation of the proliferative phase of wound healing, bridging the inflammatory and remodeling phases. These three stages are closely interconnected and inseparable. This paper comprehensively integrates the regulatory mechanisms of new blood vessel formation in wound healing, focusing on the proliferation and migration of endothelial cells and the release of angiogenesis-related factors under different healing outcomes. Additionally, the hidden link between the inflammatory environment and angiogenesis in wound healing is explored.

## 1 Introduction

A successful wound healing process hinges upon the orchestrated interplay of three distinct stages: the inflammatory phase, proliferative phase, and remodeling phase. Over the course of wound repair, the establishment of a novel vascular system unfolds as an ongoing endeavor. Within the confines of the wound site, an initial period witnesses fervent and efficient angiogenesis, culminating in the emergence of a disorganized and fledgling network of neovessels. Subsequent to this, a methodical process of pruning these neovessels transpires, with the aim of reinstating a vascular network milieu akin to its pre-injury state. However, the manifestation of pathological angiogenesis can take the form of either impaired or excessive vessel formation. The former is intricately tied to the emergence of non-healing wounds, while the latter finds correlation with the pathogenesis of conditions such as pathological scars, tumors, arthritis, and retinal disorders. Concurrently, the inflammatory phase and angiogenesis are inextricably linked, given that an array of inflammatory cells has been substantiated as sources of pro-angiogenic factors. This symbiotic relationship begets a hierarchical regulatory role for both processes within the intricate framework of wound repair.

## 2 Vascular development and regulation in tissue repair

Vascular development is a complex process involving diverse cell types and microenvironmental alterations (Schultz et al., 2011). This process encompasses key stages, including activation, sprouting, regression, and maturation (Wietecha et al., 2013). At the cellular level, endothelial cells play a pivotal role as the foundational vascular scaffold during tissue repair. In the phase of angiogenic sprouting, these cells undergo a sequence of events, including activation, adhesion, proliferation, and migration (Wacker and Gerhardt, 2011; Chen et al., 2019). On the molecular level, angiogenesis is a multidimensional process wherein existing or surviving vessels form new blood vessels, dynamically regulated by numerous cellular mechanisms and mediators (De Palma et al., 2017).

### 2.1 Stimulation and activation of neoangiogenesis

#### 2.1.1 Regulation of VEGF-Driven angiogenic signaling pathways by various signaling

FactorsVascular regeneration is primarily mediated through the signaling of Vascular Endothelial Growth Factor Receptor-2 (VEGFR-2) in endothelial cells (Simons et al., 2016). Over 30 years ago, VEGF, also known as vascular permeability factor (vPF), was discovered by Senger et al. in tumor cells. VEGF is an essential factor in angiogenesis and remodeling, mediating cell proliferation, angiogenesis promotion, and increased permeability capabilities. Growth factors and their receptors are subject to regulation by various molecules and their receptors, and they can also modulate the expression of downstream receptors and cellular behaviors. Hypoxia is a crucial driving factor for angiogenesis, and Hypoxia-Inducible Factors (HIFs) serve as the principal regulatory factors in cellular responses to hypoxia (Semenza, 2012). Under conditions of low oxygen or hypoxia, the finely orchestrated vascular homeostasis governed by prolylhydroxylase domain-containing enzymes (PHDs) becomes disrupted, leading to the accumulation of hypoxia-inducible factor-1 alpha (HIF-1α) (Salceda and Caro, 1997; Hickey and Simon, 2006). This shift propels the transition of the vasculature from quiescence to an active state, creating a favorable surrounding, for neovascularization. In hypoxic environments, various responsive and inflammatory cells release pro-angiogenic factors, with vascular endothelial growth factor (VEGF) occupying a prominent position. VEGF is closely associated with the accumulation of Hypoxia-Inducible Factor 1-alpha (HIF-1α). HIF-1α regulates downstream VEGF, and together, they play a concerted role in the process of angiogenesis (Zhang et al., 2018). Additionally, VEGF is expressed in various cell types, including keratinocytes involved in the formation of the epidermis. FOXO1, a forkhead transcription factor involved in a wide range of cellular processes, is significantly activated in the leading edge and basal layer of keratinocytes after skin injury (Jeon et al., 2018). It promotes the signal transduction of VEGF in keratinocytes by downregulating the anti-angiogenic signal CD36 (Ren, 2018). The PI3K/AKT/mTOR pathway plays a crucial role in regulating cell growth, metabolism, and biosynthesis. It modulates angiogenesis either through HIF-1α-dependent or HIF-1α-independent mechanisms, increasing the expression of VEGF and other endothelial growth factors (Zhong et al., 2000; Chen and Meyrick, 2004; Falcon et al., 2011). Simultaneously, it can promote angiogenesis by enhancing the migration of keratinocytes and fibroblasts and inducing VEGF expression (Du et al., 2023). The upregulation of VEGF leads to weakened cell-cell connections and increased microvascular permeability, marking the initiation of the activation and sprouting phases in new blood vessel formation (Weis and Cheresh, 2005). Notch serves as a direct molecular guide for tip cell development. The expression of VEGF/VEGF-R and the chemokine receptor CXCR4 are critical molecules controlling the activity of the Notch signaling pathway. Additionally, VEGF triggers the expression of the Notch ligand dll4 in tip cells, establishing a Notch-Dll4 signaling coupling to promote vascular sprouting and arterial formation (Pitulescu et al., 2017).

#### 2.1.2 Mechanical stress regulation in the early stage of angiogenesis

Clues from mechanical mechanics have been demonstrated to significantly impact angiogenic sprouting through various mechanisms, particularly in determining the location of tip cell formation (Yung et al., 2009; Barrasa-Ramos et al., 2022). Among these, shear stress generated at the apex of endothelial cells in response to intraluminal flow appears to be particularly relevant in determining the axial position of sprouting (Ghaffari et al., 2017). Intraluminal flow, at low values, promotes the degradation of the endothelial cell basement membrane by regulating matrix metalloproteinase activity, thereby altering vascular permeability (Seano et al., 2014; Fey et al., 2016). Transvascular and interstitial blood flows seem crucial in determining the circumferential position of vascular occurrence (Ghaffari et al., 2017). The transduction of mechanical signals into cellular biological signals is intricately linked to the key transcriptional regulatory factor YAP/TAZ (Totaro et al., 2018). The stretching of the cellular scaffold activates YAP/TAZ, leading to their translocation from the cytoplasm to the cell nucleus. Shear stress generated by blood flow has also been shown to transport YAP/TAZ into the nuclei of endothelial cells (Nakajima et al., 2017). Notably, the Hippo pathway, particularly the YAP/TAZ pathway, orchestrates endothelial contact inhibition and the modulation of vascular endothelial calcification protein, thereby contributing to vascular sprouting (Choi et al., 2015). The EGFR-RAS-RAF-MEK-ERK signaling axis can stimulate the activation of LATS kinase and influence downstream Hippo pathways (Vlahov et al., 2015). Furthermore, YAP/TAZ mediate VEGF-VEGFR2 signaling in angiogenesis and foster the establishment and maturation of the vascular barrier (Kim et al., 2017; Wang et al., 2017). Presently, *in vivo* imaging microscopy directly captures the formation of novel, distorted microvasculature during wound healing. This nascent vasculature exhibits heightened sprouting frequency compared to adjacent normal capillaries, likely attributed to signaling pathway activation resulting from perturbed downstream hemodynamic properties (Chong et al., 2017).

### 2.2 Selective regression of neoangiogenesis

Simultaneously, as vasculature matures, selectively regressing neovessels eventually restore the capillary density present prior to injury. Insufficient perfusion of certain newly formed capillaries induces diminished shear stress and a dearth of endothelial cell survival signals, TIE1 and TIE2, integral in physiological vessel regression (Ando and Yamamoto, 2013). During such regression, endothelial cells migrate from pruned vessels to neighboring ones due to differential shear forces (Chen et al., 2012; Franco et al., 2015). Apoptosis signals mediated by the BCL2 family proteins do not actively initiate vessel regression; rather, they facilitate vessel clearance when migration to alternative vessels is unfeasible (Franco et al., 2015; Watson et al., 2016). In pathological vessel regression, observed in conditions like diabetic retinopathy and diabetic nephropathy, hyperglycemia amplifies vessel regression and endothelial cell apoptosis. Elevated glucose levels heighten the BAX/BCL2 ratio and activate protein kinase C, with VEGF assuming a pivotal role in high-glucose-induced endothelial cell apoptosis (Quagliaro et al., 2003; Yang et al., 2008). Concomitantly, during wound healing, the regression of nascent capillaries closely involves negative vascular regulatory factors. Among these, pigment epithelium-derived factor (PEDF), a serpin family member, emerges as a promising anti-angiogenic factor within the vascular system. It is recognized for its ability to induce endothelial cell apoptosis and reduce the permeability of leaky neovessels (Aurora et al., 2010). Another influential factor in wound capillary remodeling is Sprouty-2 (SPRY2). Functioning as an intracellular protein, SPRY2 impedes the mitogen-activated protein kinase (MAPK) signal, ultimately attenuating the impact of VEGF on endothelial cell proliferation during wound healing (Cabrita and Christofori, 2008).

## 3 The intrinsic relationship between hypoxia and poor neovascularization

### 3.1 High glucose suppresses HIF-1α-mediated pro-angiogenic pathways

**FIGURE 1:**
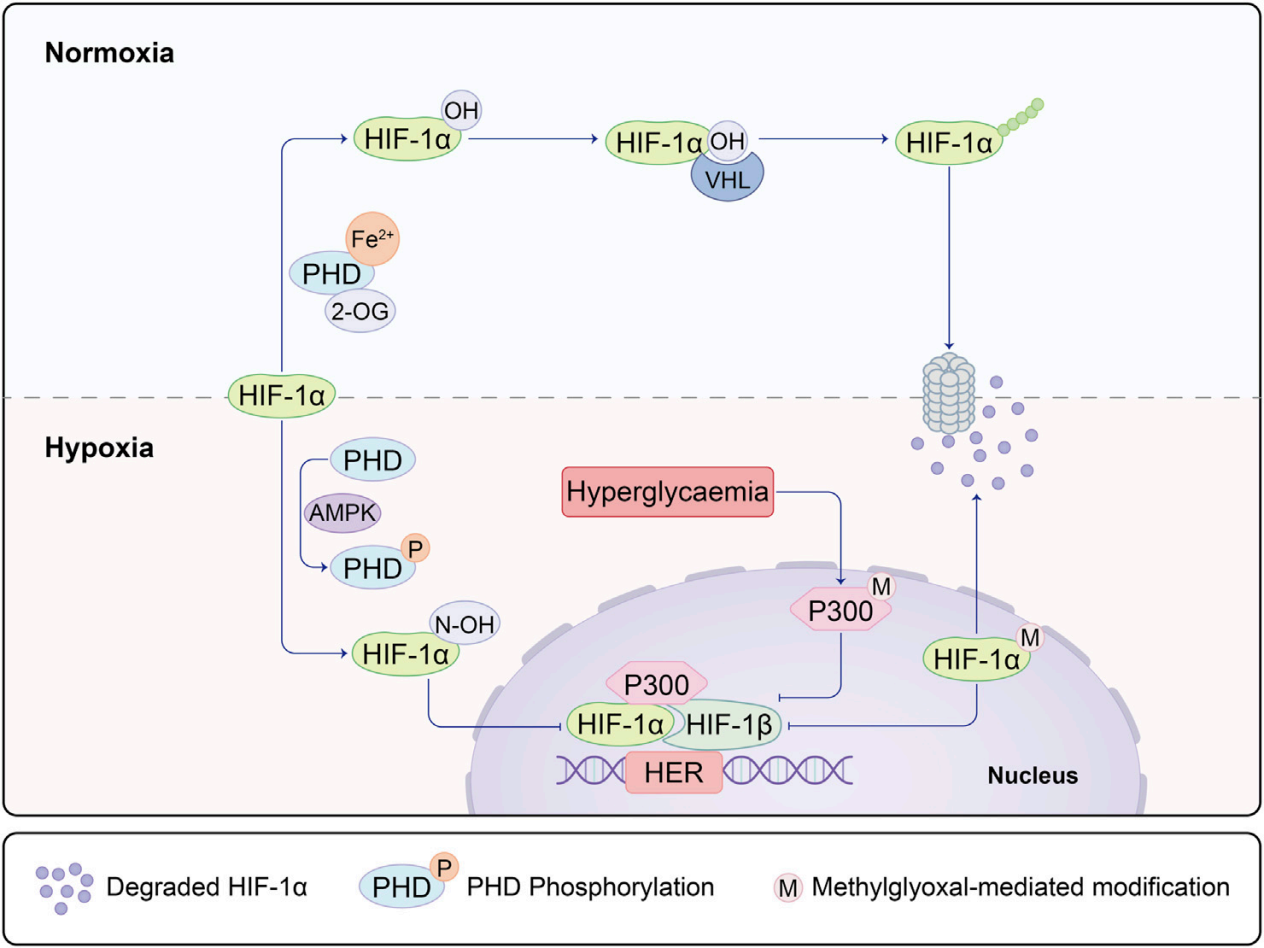
Under hypoxic and non-diabetic conditions, HIF-1α is stabilized and translocates to the nucleus, where it forms a dimer with HIF-1β on the hypoxia response element (HRE) of target genes. This complex recruits co-activators, including p300, facilitating the transcription of HIF-1 target genes. This process mediates the adaptive response to hypoxia and promotes vascularization in wound healing through the activation of the HIF-1α/VEGF signaling pathway along the regulated axis. In diabetic conditions, both the degradation of HIF-1α mediated by prolyl hydroxylase (PHD) and the modification of HIF-1α mediated by methylglyoxal are enhanced, leading to the ubiquitination of HIF-1α. Concurrently, methylglyoxal inhibits the recruitment of HIF-1 dimers and co-activators by modifying p300. This inhibition suppresses the dimerization of HIF-1 and the recruitment of co-activators, ultimately preventing the expression of HIF-1α. As a result, this molecular cascade contributes to the altered response to hypoxia in diabetic conditions.

Numerous studies underscore the association between impaired angiogenesis and delayed wound healing, with diabetes often leading to chronic non-healing wounds (Chao and Cheing, 2009). Oxygen assumes a pivotal role in nearly all aerobic cellular respiration and metabolic processes during the wound healing trajectory. Yet, both oxygen deprivation and excess can exert substantial influence on the healing dynamics. Hypoxia, instigated by oxygen deficiency, prompts the activation of hypoxia-inducible factor-1 alpha (HIF-1α). This master regulator not only influences downstream VEGF signaling but also exerts control over the HIF1α-PFKFB3 pathway. Of particular significance, PFKFB3 plays a decisive role in dictating the competition amongst endothelial tip cells and, akin to VEGF, assumes a central role in the initiation of vascular neogenesis (Min et al., 2021). Amid normoxic conditions, prolyl hydroxylase domain-containing enzymes (PHDs) transition into an active state upon encountering oxygen and divalent iron. This activation sets the stage for the hydroxylation of HIF-1α, subsequently subjecting it to ubiquitination by Von Hippel-Lindau protein (VHL), a ubiquitin E3 ligase. The ubiquitinated HIF-1α is then subjected to degradation via the 26S proteasome. However, under hypoxic circumstances, HIF-1α stabilizes and migrates into the cellular nucleus, where it dimerizes with HIF-1β. The resulting complex binds to hypoxia-response elements (HREs) nestled within HIF target genes, thereby instigating their transcription. High glucose conditions inhibit the activity of the N-terminal and C-terminal transactivation domains of HIF-1α (Botusan et al., 2008). Moreover, elevated glucose levels incite the accumulation of methylglyoxal, which destabilizes HIF-1α via PHD-independent or VHL-independent mechanisms (Ceradini et al., 2008). In scenarios marked by high glucose and hypoxia, the methylglyoxal-mediated activation of HIF-1α and the co-activator p300 mutually hinder transcriptional processes (Figure 1). Chen Z, Zhu Y, and others have also discovered that hypoxia can regulate angiogenesis, including endothelial cells, through the HIF-1α/Let-7s/AGO1/VEGF pathway. This involves negative regulation of AGO1, leading to a significant increase in VEGF protein expression (Chen et al., 2013; Zhu et al., 2020).

### 3.2 Regulation of angiogenesis by AMPK under hypoxic conditions

**FIGURE 2:**
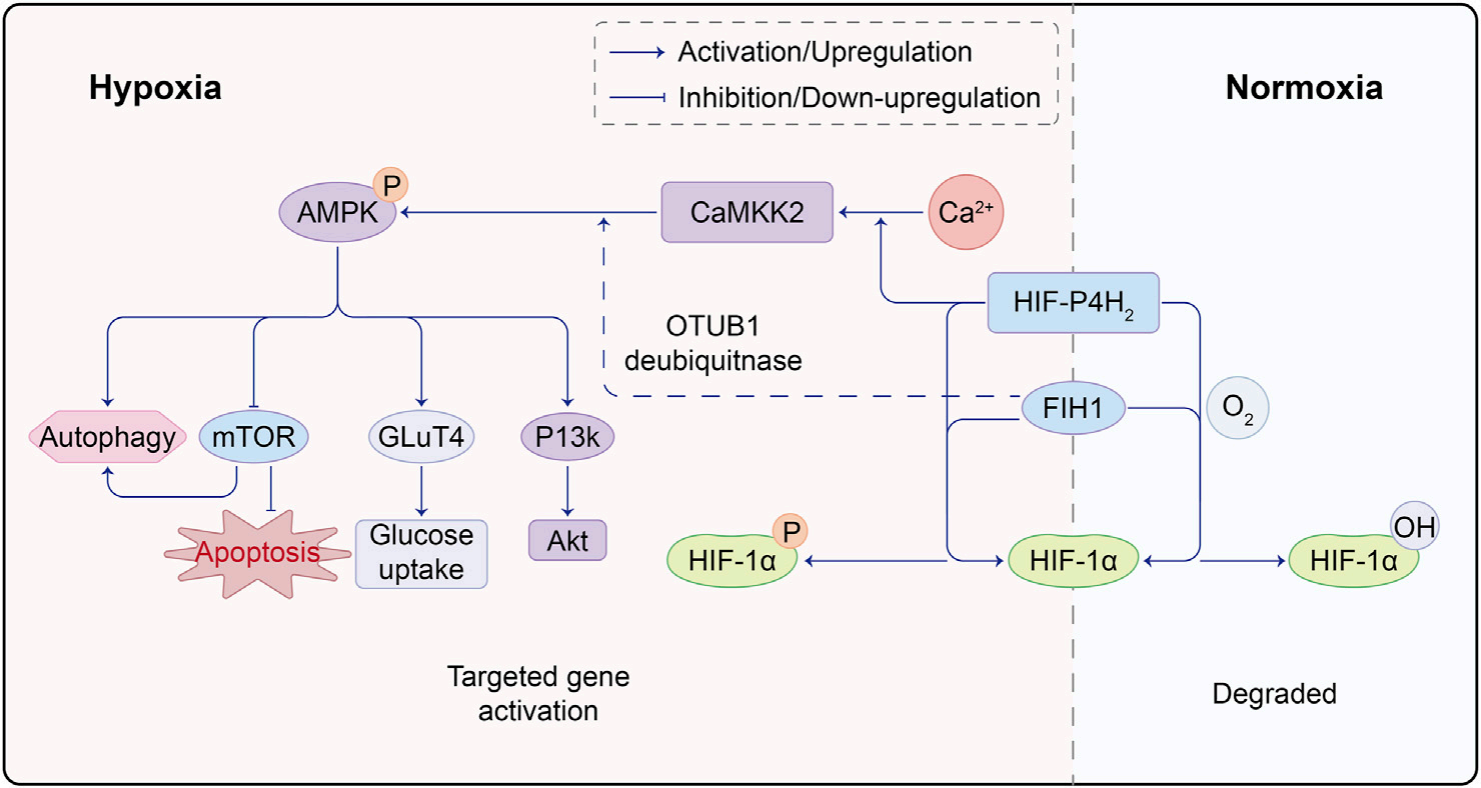
Molecular Crosstalk Between AMPK and HIF Pathways. HIF-P4H2, a protein responsible for regulating HIF, plays a crucial role. Under normoxic conditions, HIF-P4H2 hydroxylates proline residues on HIF-1α through its oxygenase activity, enhancing its binding affinity to von Hippel-Lindau (VHL) protein and marking it for degradation. However, under hypoxic conditions, reduced HIF-P4H2 activity leads to decreased HIF-1α hydroxylation, making it less prone to VHL binding, resulting in HIF-1α accumulation; FIH1 is another key protein in the hypoxic response. It inhibits the interaction between HIF-1α and transcription co-factors CBP/p300, suppressing HIF-1 transcriptional activity. Under hypoxia, FIH1 activity decreases, less effectively inhibiting HIF-1α and CBP/p300 interaction, allowing enhanced binding and increased HIF-1 transcriptional activity. Under hypoxia, HIF-P4H2 binds Ca, promoting downstream CaMKK2/AMPK activation. Simultaneously, FIH1 can interact with ubiquitin ligase OTUB1, facilitating AMPK activation. This intricate molecular interplay underscores the complex crosstalk between HIF and AMPK pathways, with HIF-P4H2 and FIH1 playing key roles in mediating their interaction. Such molecular interactions offer vital insights into adaptive mechanisms in low oxygen environments.

In recent years, the role of glucose and lipid metabolism in angiogenic sprouting has gained attention. AMP-activated protein kinase (AMPK) has long been recognized for its role in regulating glycolysis and fatty acid oxidation. It has been used to treat diabetes by inducing glucose uptake in muscle cells to expend energy. The activation of CaMKK/AMPK can regulate glucose uptake and metabolism in diabetes treatment (Entezari et al., 2022). AMPK stimulates the phosphorylation of two crucial regulatory proteins, TBC1D1 and TBC1D4, of glucose transporter 4 (GLUT4), promoting glucose uptake by muscle cells (Spaulding and Yan, 2022). AMPK has been shown to have both positive and negative regulatory effects in angiogenesis. Under physiological conditions such as hypoxia, local ischemia, and exercise, activated AMPK signaling inhibits mTORC1 signaling, inducing cellular autophagy. The AMPK/mTOR pathway prevents oxidative stress in the endoplasmic reticulum and mitochondria caused by high glucose, protecting endothelial cells from apoptosis and dysfunction (Varshney et al., 2017; Jin et al., 2018). Some studies also suggest that endothelial cell autophagy stabilizes HIF-1α, promoting vascular expression (Salminen et al., 2016). Stimulating AMPK phosphorylation also activates the PI3K/AKT axis, controlling inflammation and downstream mTOR activation to promote angiogenesis. However, under pathological and tumor conditions, such as in diabetic retinopathy, high glucose can inhibit AMPK activity and activate mTOR. The latter upregulates HIF-1α, leading to increased VEGF expression. Emerging evidence implies that AMPK activation in tumor cells engenders the ubiquitination and degradation of HIF-1α, thereby counteracting its activation (Seo et al., 2016; Wang et al., 2022a; Chen et al., 2022). Notably, Cheng Wang and colleagues postulate the coexistence of PHD2 and AMPK within the same complex. AMPKα1 phosphorylates PHD2, rendering it inactive and curtailing the PHD2-mediated hydroxylation and degradation of HIF-1α (Wang et al., 2022b). The profound interrelation between AMPK-dependent and -independent regulation of HIF-1α, as well as the potential cross-linkage between these pathways, remains a terrain ripe for further exploration (Figure 2).

### 3.3 Impaired macrophage function in chronic wounds

Macrophages are crucial cells derived from the immune system during the repair process. In fact, macrophages play a pivotal role in the early stages of angiogenesis by guiding the sprouting of endothelial cells. Macrophages establish crosstalk with endothelial cells during angiogenesis by producing various growth factors such as VEGF, PDGF, fibroblast growth factor (FGF), and TNF. Additionally, matrix metalloproteinases (MMPs) produced by macrophages assist in creating a biologically useable gradient of VEGF, guiding endothelial sprouting (De Palma et al., 2017). Recently, through *in vivo* imaging, it has been discovered that macrophages also occupy the gaps between the tips of blood vessels in skin wound healing. Macrophages around newly formed vessels contribute to the anastomosis of vessels and the stability of late-stage angiogenesis (Blanco and Gerhardt, 2013). However, in diabetic wounds, the increase in advanced glycation end products (AGEs) due to high glucose leads to inherent sensitivity of marrow progenitor cells in diabetic mice to pro-inflammatory macrophage polarization. Although their polarization towards pro-inflammatory M1 phenotype is heightened, their phagocytic activity is significantly reduced, resulting in the accumulation of neutrophils in diabetic wounds (Ishida et al., 2019). Simultaneously, this interferes with their polarization towards the anti-inflammatory, pro-healing M2 phenotype (Pavlou et al., 2018).

Macrophages affected by hyperglycemic environment consequently discharge elevated levels of pro-inflammatory cytokines, thereby instigating a decline in the population of M2 phenotypic cells while ushering in an upswing in the proportion of M1 phenotypic cells (Mirza and Koh, 2011). Furthermore, emerging research underscores the role of inflammatory macrophages in the initial stages of wound repair initiation, wherein they orchestrate the disintegration of neutrophils within wounds. The untimely inhibition of TNF-α expression can potentially undermine neovascularization (Gurevich et al., 2018). Therefore, the reduced angiogenesis in diabetic wounds may be related to macrophage dysfunction.

### 3.4 The influence of microRNA on angiogenesis

An increasing body of evidence indicates that non-coding RNA, particularly microRNA, can either promote or inhibit endothelial cell proliferation, migration, and lumen formation, ultimately influencing angiogenesis (Bartel, 2004; Voellenkle et al., 2012). Furthermore, an array of data substantiates the notion that the dysregulation of miRNA-mediated angiogenesis regulation contributes to an array of maladies, encompassing vascular tumors, tumor development, and aortic dissection. In the recent years, research inquiries have ventured into the realm of miRNAs and their contribution to angiogenesis within the context of chronic non-healing wounds. MiRNAs, through their capacity to target and regulate RNA and protein synthesis, exert modulatory influence over cellular biological effects and angiogenic factors, thus wielding considerable sway over the intricacies of angiogenesis in non-healing wounds. Pertinently, inhibitors of miRNA-200b evince a propensity to enhance angiogenesis, with their diminished expression aligning with hypoxic conditions and a concomitant reduction in HIF-1α expression (Chan et al., 2011). In a downstream progression, Ets-1 emerges as a novel target—a transcription factor with a stake in angiogenesis. Notably, Ets-1 steps in to salvage miRNA-200b-induced detriment, stymieing the trauma-induced process of angiogenesis inhibition. This serves as a compelling illustration that transient miR-200b downregulation serves as an initiator of the angiogenic cascade. Moving to the diabetic wound context, the expression of miRNA-200b impinges upon the expression of GATA2 and VEGF2, leading to a constriction of angiogenesis (Chan et al., 2012). In a converse vein, the quelling of miR-200b expression fosters the resurgence of Notch1 expression and reactivates the Notch pathway, thereby amplifying the growth trajectory of vascular endothelial cells (Qiu et al., 2021). Moreover, miRNA-210, elicited by hypoxia, precipitates heightened expression of the Notch1 signaling molecule, a stimulus that precipitates the migration of endothelial cells and augments the processes of angiogenesis (Lou et al., 2012). However, in a contrasting realm, miR-20b-5p assumes a role in tempering the Wnt9b/β-catenin signaling pathway, consequently dampening endothelial cell function and putting a check on angiogenesis (Xiong et al., 2020).

## 4 Pathological excessive angiogenesis and scar formation

In certain pathological contexts, the upregulation of angiogenesis assumes a pivotal role as a pathological hallmark driving the onset and progression of diseases. Malignant tumors stand as quintessential examples, relying heavily on neovascularization for an unceasing supply of nutrients that facilitate tumor proliferation, invasion, and metastasis. Moreover, the trajectory of scar formation is intrinsically linked to excessive angiogenesis within the healing process of superficial skin wounds (Korntner et al., 2019).

### 4.1 The role of endothelial cells in scar formation

This aspect comes to the fore during the emergence of hyperproliferative scars and hypertrophic scar nodules, marked by a considerable elevation in capillary content and neovascularization responses, surpassing the usual baseline levels (Amadeu et al., 2003; van der Veer et al., 2011). Low-apoptotic myofibroblasts (Hmyos) also contribute to the process of angiogenesis. Hmyos produce signal entities called microvesicles, significantly increasing three cellular processes of angiogenesis: endothelial cell proliferation, migration, and assembly into capillary-like structures (Laberge et al., 2021). On one facet, the incomplete structural makeup of nascent blood vessels during angiogenesis bestows upon them elevated permeability, facilitating immune cell infiltration and the entry of inflammatory cytokines from microvessels into the extracellular matrix (ECM). This results in heightened local inflammation and promotes the formation of hypertrophic scars (Ogawa and Akaishi, 2016). Moreover, fibronectin in the exudate serves as a substrate for fibroblast attachment and inward growth, providing the matrix. The implications of this are underscored by the work of Christian et al., who have delineated that, during the emergence of glial scars within the nervous system, fibrinogen functions as an early signal within the TGF-β/Smad pathway (Schachtrup et al., 2010). Upon the infiltration of fibroblasts, collagen deposits are precipitated. Conversely, the signals emanating from apoptotic endothelial cells within poorly perfused neovessels, stemming from inadequate blood flow, set the wheels in motion for the development of fibrosis. *In vitro* studies have provided compelling evidence, demonstrating that the conditioned media sourced from apoptotic endothelial cells elicits heightened local fibroblast adhesion and prompts the augmented expression of α-smooth muscle actin, effectively serving as an indirect contributor to the process of scar formation (Chang et al., 2022).

Furthermore, endothelial cells in scar nodules can lose their original adhesive characteristics and apical-basal polarity, transforming into migratory undifferentiated mesenchymal cells that invade adjacent tissues. This differentiation process is known as endothelial-to-mesenchymal transition (EndoMT) (Medici and Kalluri, 2012). Fibroblasts and myofibroblasts in scar nodules are derived from endothelial cells. Matsumoto et al. and Tanaka et al. observed a significant increase in the expression of pro-inflammatory and pro-fibrotic genes, SERPINA3 and LAMC2, and the vascular generation-related gene VEGF in CD34 + vascular endothelial cells within scar tissue (Matsumoto et al., 2020; Tanaka et al., 2019).

### 4.2 Extracellular matrix in pathological scarring

In the process of angiogenesis during wound healing, the degradation of extracellular matrix (ECM) promotes vascular sprouting, with endothelial tip cells migrating into the tissue to provide a conducive environment. However, the degradation of ECM components in this process may stimulate compensatory collagen production by neighboring fibroblasts, leading to increased deposition of scar tissue. Several studies have suggested that reducing vascular production can effectively alleviate scar healing, potentially by altering the ECM homeostasis and improving the state of damaged degradation and excessive accumulation (Ferguson et al., 1996). In hypertrophic scar (HTS) formation, there is an imbalance in ECM synthesis and remodeling (Ulrich et al., 2010). Fibroblasts and myofibroblasts persist due to apoptosis defects, resulting in sustained presence of myofibroblasts, excessive deposition of fibroblast collagen I, and scar formation (Sidgwick and Bayat, 2012). Nodules containing myofibroblasts are characteristic of hypertrophic scars (Xue and Jackson, 2015). MMP1, a matrix metalloproteinase (MMP) that can improve scar formation, is downregulated during HTS formation, leading to reduced degradation of ECM components such as collagen I, collagen III, and fibronectin. In hypertrophic scar nodules, collagen production is 20 times higher than normal scars, and the ratio of type I to type III collagen (17:1) is three times higher than in normal scars (6:1). Scar nodules lack elastic fiber, hyaluronic acid, and elastin, resulting in stiff scar tissue (Sidgwick and Bayat, 2012).

### 4.3 Scarless healing in fetal wounds

During the course of human fetal development, a remarkable phenomenon called scarless wound healing prevails, albeit exclusively in the early stages of fetal growth. Subsequent to around 24 weeks of human fetal development or approximately 16–18 days of mouse gestational age, the transition from scarless wound healing to scar-forming commences. The mechanisms and processes governing scarless wound healing in fetuses offer a realm of novel insights into potential avenues for the prevention and treatment of scars in adults (Walmsley et al., 2015; Shakoor et al., 2021) (Cass et al., 1997). Comparative analysis between fetal and adult repair models has revealed that scarless wounds exhibit lower levels of VEGF compared to their scar-forming counterparts. Remarkably, the exogenous introduction of VEGF is capable of converting a scarless wound model into one that forms scars (Wilgus et al., 2008). This notion is corroborated by animal repair models, where the inhibition of angiogenesis yields enhanced healing outcomes. This manifests through the suppression of fibroblast proliferation and collagen deposition (Li et al., 2009), the reduction of vascular density, and the contraction of scar width (Wilgus et al., 2008). Furthermore, scientific inquiry has unveiled that curtailing redundant and non-functional neovascularization can foster wound healing while simultaneously minimizing scar formation. The expeditious pace of wound healing on oral mucosa, in contrast to skin wounds, is aligned with the relatively weaker angiogenic response in oral mucosa. Furthermore, upon juxtaposing the scarless and scar-forming phases of fetal repair, it emerges that VEGF expression experiences a substantial upsurge during the scarless repair stage. However, it is intriguing to observe that the receptors for VEGF, namely, VEGFR-1 and VEGFR-2, undergo a downregulation of around 30%–50% in all scarless repair fetal mice when compared to the skin of age-matched control mice. Notably, this downregulation does not culminate in a significant discrepancy in the quantity of blood vessels between the two groups (Colwell et al., 2005). This phenomenon hints towards the possibility that the diminution of VEGFR-1 and VEGFR-2 could potentially mitigate unnecessary non-functional vascular perfusion, thereby exerting control over scar formation. Consequently, it can be inferred that once the angiogenic response surpasses the optimal threshold required for effective wound healing, the onset of scar formation becomes inevitable. Research pertaining to the transplantation of exosomes sourced from fetal mesenchymal stem cells, aimed at promoting scarless wound healing in diabetic wounds, further underscores the pivotal role played by fetal mesenchymal stem cells in the context of scarless wound healing (Wang et al., 2022a). The metabolic state of adult mesenchymal stem cells tends to veer towards aerobic glycolysis, while fetal cells demonstrate a propensity towards relying on oxidative phosphorylation as their primary energy source. Pre-existing research by B. WANG et al. has lent credence to the notion that the introduction of fetal cell mitochondria triggers metabolic reprogramming in adult cells. This metabolic state of mesenchymal stem cells is intertwined with the phenomenon of cellular senescence. Furthermore, the presence of a less acidic microenvironment also assumes significance in the landscape of wound healing (Shakoor et al., 2021). Notably, within the spectrum of fetal scarless healing, a substantial concentration of glycosaminoglycan hyaluronic acid (HA) is discernible within the fetal extracellular matrix. This abundance of HA, in turn, augments the proliferation and migration of fibroblasts (Longaker et al., 1991). By contrast, hyaluronic acid assumes a transient role in the early stages of adult wound healing. In the context of rabbit fetuses, the degradation of hyaluronic acid precipitates heightened collagen production and neovascularization. Leveraging this contrast, B. A. Mast et al. ventured to hypothesize that the active components stemming from the degradation of hyaluronic acid contribute to the adult wound healing response. Their investigation culminated in the discovery that treatment with hyaluronic acid degradation products yielded a marked increase in collagen content and neovascular response compared to the control group. This substantiates the notion that hyaluronic acid degradation products indeed play a contributory role in the process of scar formation.

## 5 Interaction between inflammatory response and angiogenesis

Vascularization is a highly integrated, multicellular process that relies on various cell activities within the microvascular environment, with the crucial involvement of immune cells. The interplay between angiogenesis and the onset of inflammation plays a synergistic role in wound healing. Various inflammatory cells such as neutrophils, monocytes, lymphocytes, macrophages, among others, release multiple angiogenic factors upon exiting the bloodstream. These factors, including vascular endothelial growth factor (VEGF), stimulate endothelial cells to produce adhesion molecules, recruiting inflammatory cells to leave the bloodstream, thereby forming a feedback loop. Simultaneously, angiogenesis maintains the inflammatory state by supplying oxygen and nutrients to the inflammatory site, providing a substantial surface area for the production of necessary cell factors, adhesion molecules, and other inflammatory mediators. However, when chronic inflammation or inadequate clearance of pathogens leads to the failure of the inflammatory response, the increased blood flow to the inflamed tissue is necessary to sustain the survival of inflammatory cells producing these factors. In this context, inhibiting the growth of new blood vessels may potentially control the inflammatory response, presenting an avenue for preventing and treating chronic inflammatory diseases (Gallo, 2000; Zittermann and Issekutz, 2006; Costa et al., 2007).

### 5.1 ANG-TIE—Key mediators in inflammation and vascularization

**FIGURE 3:**
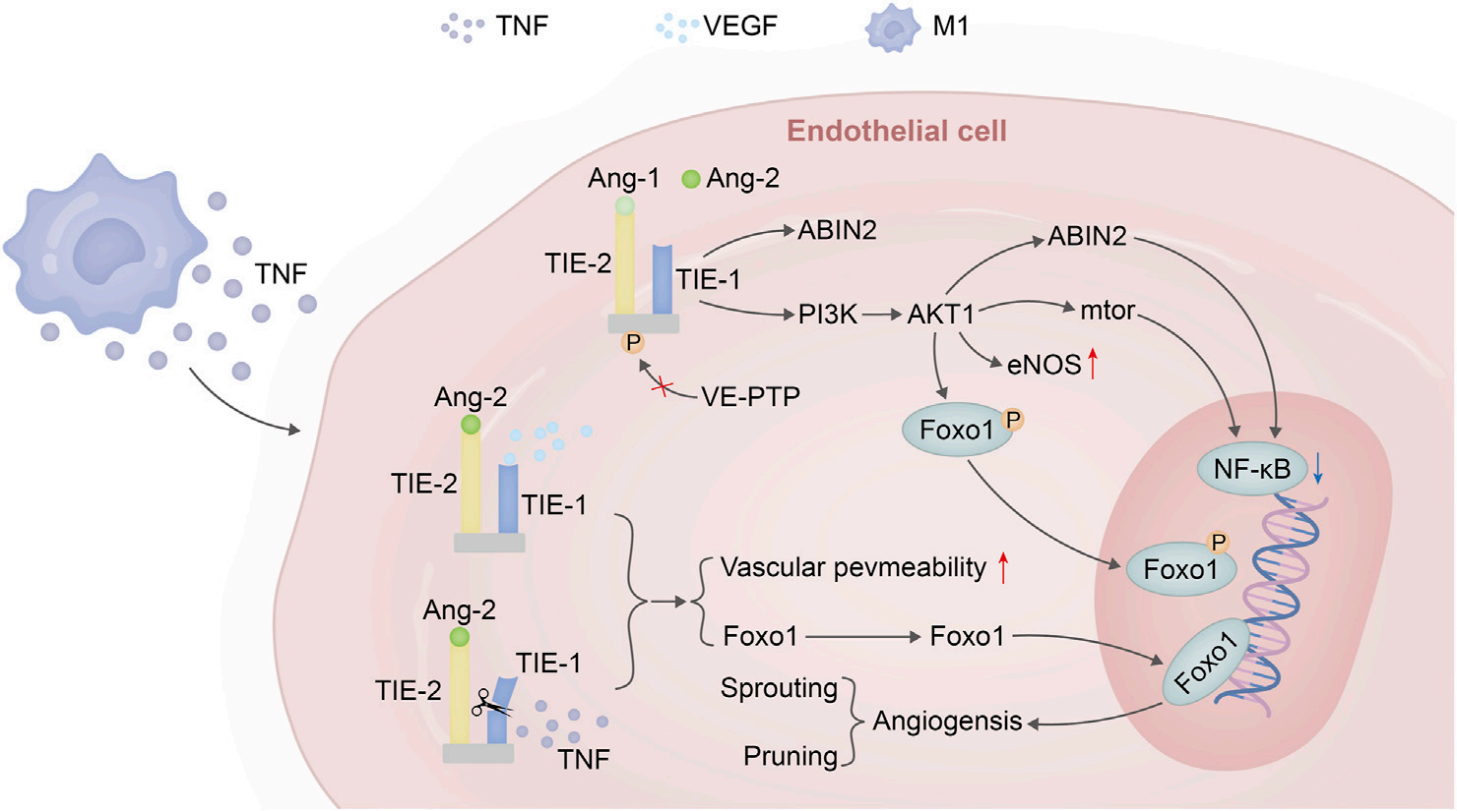
Angiopoietin–TIE signalling in endothelial cells in homeostasis versus inflammation versus VEGF. Under steady-state conditions, the binding of Ang-1 to the TIE2 receptor leads to the phosphorylation of TIE2, activating the PI3K/AKT1 pathway. This pathway phosphorylates FOXO1, triggering nuclear exclusion of this transcription factor followed by degradation. Additionally, it promotes eNOS to maintain vascular homeostasis and can further enhance downstream mTOR expression or induce ABIN2 to suppress the expression of key factors in the NF-κB inflammatory pathway. In the presence of inflammation and sufficient VEGF expression, when TNF is released by M1 macrophages, the extracellular domain of the TIE1 receptor is cleaved. Due to the dominant antagonistic activity of ANGPT2 over agonists, TIE2 remains unphosphorylated. Consequently, FOXO1 functions as a transcription factor leading to the expression of genes associated with vascular instability (sprouting) and cellular apoptosis (pruning).

The ANG-TIE receptor pathway assumes significance in the realms of vascular permeability and pathological vascular remodeling, thus underscoring the intersection between inflammation and this process (Saharinen et al., 2017). TIE1, with its role as an ANG2 converter, is subject to cleavage by TNF released during inflammation. This culminates in the antagonistic activity of ANG2, rendering TIE2 incapable of phosphorylation and allowing FOXO1 to execute its functions unhindered. This eventuality leads to a reduction in vascular stability and a simultaneous enhancement of vascular permeability, thereby creating an environment conducive to neovascular sprouting (Kim et al., 2016; Eklund et al., 2017). In a similar vein, the VEGF discharged by inflammatory cells can activate TIE2’s tyrosine kinase activity through the cleavage of the extracellular domain of TIE1 (Du Cheyne et al., 2020). As the inflammatory response wanes and VEGF release subsides, the ANGPT-TIE receptor pathway undergoes pruning and a reconfiguration of mechanisms associated with endothelial cells. This encompasses responses to shear stress mediated by VE-PTP, the migration of cells within the endothelial cell-matrix milieu, inter-endothelial cell connections, and the binding of ANG1 to TIE2, an event that triggers NF-κB—a regulator of inflammatory responses. The decreased release of these factors, coupled with significant eNOS activation, collectively contribute to the well-established vascular protective function of this pathway (Figure 3).

### 5.2 Macrophages: crucial participants in the interplay of vascularization and inflammation

Macrophages emerge as pivotal orchestrators at the crossroads of angiogenesis and inflammation, owing to their versatile and adaptable phenotypes. These cells are proficient at releasing an array of dynamic cytokines, growth factors, and proteinases that wield the capacity to sculpt the local milieu. This attribute underscores their profound importance in this intricate interplay (Wynn et al., 2013). Macrophages are adept at directly instigating wound angiogenesis through the secretion of pro-angiogenic factors like VEGF. Indirectly, they influence angiogenesis by releasing cytokines such as IL-8 and proteases like MMP9, which activate blood vessels and modify the extracellular matrix, thereby fostering an environment conducive to neovascularization (Zajac et al., 2012; Alraouji and Aboussekhra, 2021; Nueangphuet et al., 2021). In recent years, the conventional dichotomy of macrophages into discrete M1 and M2 phenotypes has undergone reevaluation, with the understanding that macrophages exist along a continuum where they can concurrently or sequentially exhibit both phenotypes (Houser et al., 2011). Macrophages not only participate in angiogenesis through paracrine secretion but also, during the budding stage of angiogenesis, guide the fusion of endothelial tip cells through adhesion molecules CDH5 and PECAM1, forming a partnership with endothelial cells (Fantin et al., 2010; Liu et al., 2016). VEGF has been found to promote the polarization of M2 macrophages (Wheeler et al., 2018). Macrophages’ participation in angiogenesis extends beyond their paracrine secretions; they function as companions during angiogenesis, facilitating endothelial cell fusion in the sprouting phase. Observations indicate that macrophages located near the terminal endothelial cells express tumor necrosis factor (TNF), which suggests an M1 phenotype. Strikingly, the absence of macrophages in mice led to the development of abnormal and underdeveloped vascular systems in the testes, even though the overall endothelial cell counts remained unaffected (DeFalco et al., 2014). Furthermore, macrophages exhibiting an M2-like phenotype induce endothelial cell apoptosis and engage in the phagocytosis of apoptotic endothelial cells, thereby underpinning their role in vascular remodeling and prunning processes (Gurevich et al., 2018). Furthermore, M2-like macrophages have been implicated in controlling vascular permeability through the phosphorylation of vascular endothelial (VE)-cadherin. Their influence on the vascular barrier involves the downregulation of VLA4, which triggers a cascade of signaling involving VCAM1/RAC1/ROS/p-PYK2/p-VE-cadherin, thereby contributing to the regulation of vascular integrity (Zhang et al., 2021). Despite the ANGPT-TIE signaling system’s primary focus on endothelial cells, a noteworthy fraction of macrophages also express TIE receptors. Interestingly, this phenomenon seems to be independent of macrophage polarization states and phenotypes. The precise role of the ANGPT-TIE pathway in the migration and recruitment of macrophages to inflammatory sites remains a matter of contention. Ang-1 has the ability to induce monocyte chemotaxis through a mechanism that operates apart from Tie-2 and integrin binding. This effect is mediated by phosphatidylinositol 3-kinase and heparin (Scholz et al., 2011; Srivastava et al., 2014).

## 6 Conclusion

In conclusion, angiogenesis stands as a pivotal orchestrator in the complex symphony of wound healing and scar formation. Striking a delicate balance in angiogenic processes is paramount, as both excessive and insufficient angiogenesis can yield pathological outcomes, impacting not only the quality of life but also carrying potential life-threatening risks. The intricate interplay between angiogenesis and inflammatory responses, characterized by the participation of immune cells and the orchestration of cytokines, constitutes a fundamental aspect of wound repair. Fetal scarless wound healing presents a remarkable avenue for garnering insights applicable to scar treatment in adult individuals. In this paradigm, factors like angiogenesis and hyaluronic acid operate in concert to contribute to the overall healing process. A comprehensive grasp of the multifaceted role of angiogenesis in wound healing and scar formation lays the foundation for the development of innovative therapeutic modalities and preventive strategies. Interventions aimed at accelerating vascularization can potentially ameliorate inflammatory reactions and expedite wound healing timelines. The trajectory of future research should delve into unraveling the intricate cross-talk between diverse cell types, molecular signaling pathways, and intricate networks within angiogenesis. This pursuit aims to unearth optimal strategies that facilitate scarless wound healing. It is noteworthy that scar healing exhibits a heightened angiogenic response compared to non-scar healing, underscoring the importance of in-depth exploration into the regulatory machinery governing angiogenesis. Such insights hold the promise of not only enhancing wound healing but also averting scar formation, thus presenting an exciting avenue for therapeutic advancement.
